# 97. A Cross-kingdom Vaccine Protects against Multiple Healthcare-associated Infections

**DOI:** 10.1093/ofid/ofac492.175

**Published:** 2022-12-15

**Authors:** Shakti Singh, Ashley M Barbarino, Eman Youssef, Teklegiorgis Ghbremariam, Sunna Nabeela, Sondus Alkhazraji, Ashraf S Ibrahim

**Affiliations:** The Lundquist Institute at Harbor-UCLA Medical Center, Torrance, California; The Lundquist Institute at Harbor-UCLA Medical Center, Torrance, California; The Lundquist Institute at Harbor-UCLA Medical Center, Torrance, California; The Lundquist Institute at Harbor-UCLA Medical Center, Torrance, California; The Lundquist Institute at Harbor-UCLA Medical Center, Torrance, California; The Lundquist Institute at Harbor-UCLA Medical Center, Torrance, California; The Lundquist Institute at Harbor-UCLA Medical Center, Torrance, California

## Abstract

**Background:**

Rise in multi-drug-resistance (MDR) among pathogens and increasing populations at risk for these infections raises the threat of nearly untreatable infectious diseases. Thus, novel vaccine strategies to prevent and/or treat MDR pathogens would benefit global health immensely. *Candida albicans* (*CA*) cell wall proteins Als3p and Hyr1p protect against candidemia due to *CA* and MDR *C. auris* (*CAU*). Further, Hyr1p shares structural homology with conserved hemagglutinin (FhaB) and OmpA proteins in many Gram-negative bacteria (GNB) *Acinetobacter baumannii* (*AB*)*, Klebsiella pneumoniae* (*KP*), and *Pseudomonas aeruginosa* (*PA*). Thus, Hyr1p antigen-based active and passive vaccinations protect against *AB* and *KP* pneumonia. We hypothesized that a dual Als3p/Hyr1p antigen vaccine formulated with CAF01 (a clinical stage adjuvant) that can promote a balanced Th1/Th2/Th17 immune response is likely to protect against multiple healthcare-associated infections caused by *Candida* species and MDR GNB.

**Methods:**

To formulate Als3p/Hyr1p antigen, we mixed CAF01 with Als3p and Hyr1p antigens in the following ratios: 0/0, 10/10, 10/30, 30/10 and 30/3 µg for each dose. CD-1 mice with each dose subcutaneously, followed by booster immunization on day 21. For efficacy evaluation, vaccinated mice (n=20 mice/group) were immunosuppressed by administration of cyclophosphamide and cortisone acetate on day -2 and +3, relative to infection with either *CAU, AB*, *KP or PA*. Infection occurred two weeks following the booster immunization. For, *CA* infection, immunocompetent mice were infected intravenously two weeks following a primary booster or after a second booster given on day 35.

**Results:**

All Als3p/Hyr1p formulations induced robust immunity against both antigens. For *CA* infection, two booster immunizations provide superior protection over one booster immunization. For *CAU* and GNB, one booster dose was enough to provide significant protection. Further, both 30/10 and 10/10 vaccine formulations protected significantly against all five infections. Specifically, for fungal infections, 30/10 and 10/10 formulations showed 30-40% and 40-50% survival efficacies (*vs*. 0% for placebo), respectively.

TABLE 1. Survival efficacy of Als3p/Hyr1p formulations against Candida species and Gram-negative bacterial infections.

**Figure:**
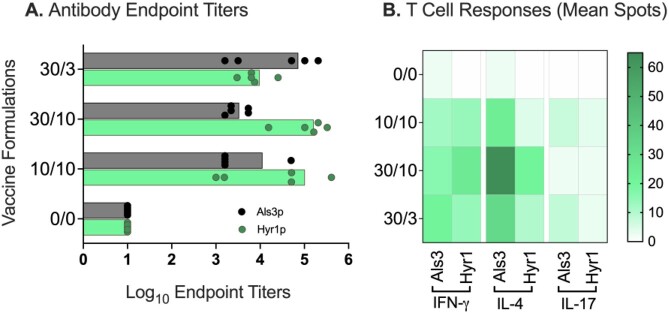

**Figure:**
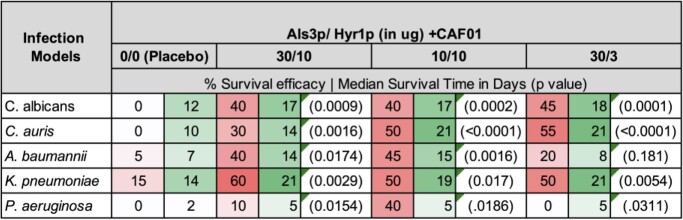

**Conclusion:**

Our study shows that the Als3p/Hyr1p induced a robust protective immunity against *CA,* MDR C*AU* and GNB.

**Disclosures:**

**All Authors**: No reported disclosures.

